# Supplementary material for: Serum SARS-CoV-2 Antigens for the Determination of COVID-19 Severity
Source: Viruses. 2022 Jul 28;14(8):1653. doi: 10.3390/v14081653 (PMC9415151; doi:10.3390/v14081653)
Supplement: Supplementary file 1 [file viruses-14-01653-s001.zip › viruses-1757499-supplementary.pdf]

Supplementary Material

<u>**Supplemental Table S1:**</u> **Demographic data of the included population.** [1] WHO clinical progression scale of 1; [2] WHO clinical progression scale of 2-3; [3] WHO clinical progression scale of 4-5; [4] WHO clinical progression scale of 6-10.

**Demography**	
**Participants (n)**	**90**
**Age (median [min-max])**	**77 [20-97]**
**Females (n [%])**	**44 [48.9%]**
Age (median [min-max])	*78 [20-97]*
**Males (n [%])**	**46 [51.1%]**
Age (median [min-max])	*77 [33-94]*
**Clinical status**	
**Asymptomatic (n [%])[1]**	11 [12.2%]
**Symptomatic (n [%])**	79 [83.3%]
Mild disease (n [%])[2]	*17 [18.9%]*
Moderate disease (n [%])[3]	*47 [52.2%]*
Severe disease (n [%])[4]	*15 [16.7%]*

Supplemental Table S2: Description of patients with discordant results between Simoa and iFlash. Red layout corresponds to negative results considering optimized cut-offs (0.099 pg/mL for Simoa and 0.31 COI for iFlash).

Patient	Gender	Age (years)	Days since symptoms	Disease category	Simoa N antigen (pg/mL)	iFlash N antigen (COI)	Spike IgG (ng/mL)	NP RT-PCR (Ct)
1	Woman	84	15	Moderate	2,59	0,37	3038	23,9
			16		1,66	0,33	5137	
			20		0,52	0,22	3479	
			22		0,099	0,19	3832	
			26		0,099	0,19	5397	
2	Man	81	15	Moderate	2697,86	159,83	13	20,1
			16		9317,69	193,92	38	
			17		1038,9	142,86	60	
			20		263,87	58,95	1518	
			21		207,42	42,19	1473	
			22		70,06	11,44	3490	
			23		25,8	3,13	7885	
			24		6,64	0,73	17550	
			26		0,32	0,18	NA	
			28		0,099	0,16	23014	
			30		0,099	0,14	27760	
			35		0,099	0,14	35277	
3	Woman	58	0	Mild	0,099	0,33	2455	34,5
4	Woman	74	2	Moderate	6,36	0,94	13805	29,2
			3		2,86	0,81	38357	
			4		2,22	0,57	31711	
			6		1,32	0,3	36794	
5	Man	81	2	Severe	4805,94	370,58	43	15,4
			3		4821,62	309,73	30	
			5		21522,48	2087,61	25	
			11		2906,28	368,26	426	
			15		30,7	3,85	3782	
			19		4,62	0,11	NA	
6	Woman	81	2	Moderate	30,88	7,99	50	14,7
			3		43,69	3,89	29	
			4		48,13	4,8	35	
			7		30,88	0,23	12	
			10		27,22	4,71	29	
7	Woman	66	8	Severe	13226,94	565,17	16	25,6
			9		15438,59	751,42	21	
			11		21765,72	226,07	1052	
			12		487,46	51,59	2311	
			14		64,69	5,01	18003	
			15		34,54	2,58	33439	
			18		2,18	0,29	59823	
			20		0,099	0,2	1178127	
8	Man	78	7	Moderate	489,02	59,04	46	22,4
			9		203,57	36,62	5755	
			12		8,31	2	30262	
			16		0,099	0,53	108151	
			20		0,099	0,54	73148	
9	Woman	94	7	Moderate	70,15	5,62	69	16,8
			8		84,88	6,77	NA	
			11		645,41	40,91	43	
			16		19,62	1,99	615	
			18		1,79	0,3	1691	
			23		0,62	0,27	2913	
			25		0,099	0,16	1815	

<u>Supplemental Figure S1:</u> Repartition of blood samples per patient per disease category.

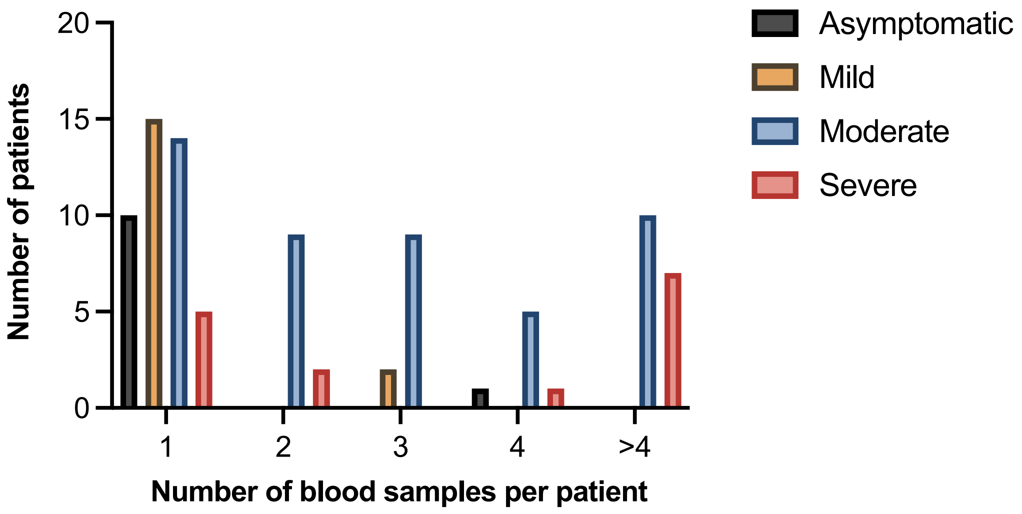

<u>Supplemental Figure S2:</u> ROC curves for cut-off determination according to different samples inclusion settings. Three different inclusion settings were used: a) earliest antigenemia value from symptom onset within the day 2 to day 14 window for a particular patient (in green), b) mean of all antigenemia values of samples collected within the day 2 to day 14 window for a particular patient (in blue) and c) the maximal antigenemia value obtained within the day 2 to day 14 window (in red). For all these analyses, 84 samples were included (n = 71 for non-severe patients and n = 13 for severe patients). These analyses were performed for the Simoa and the iFlash assays.

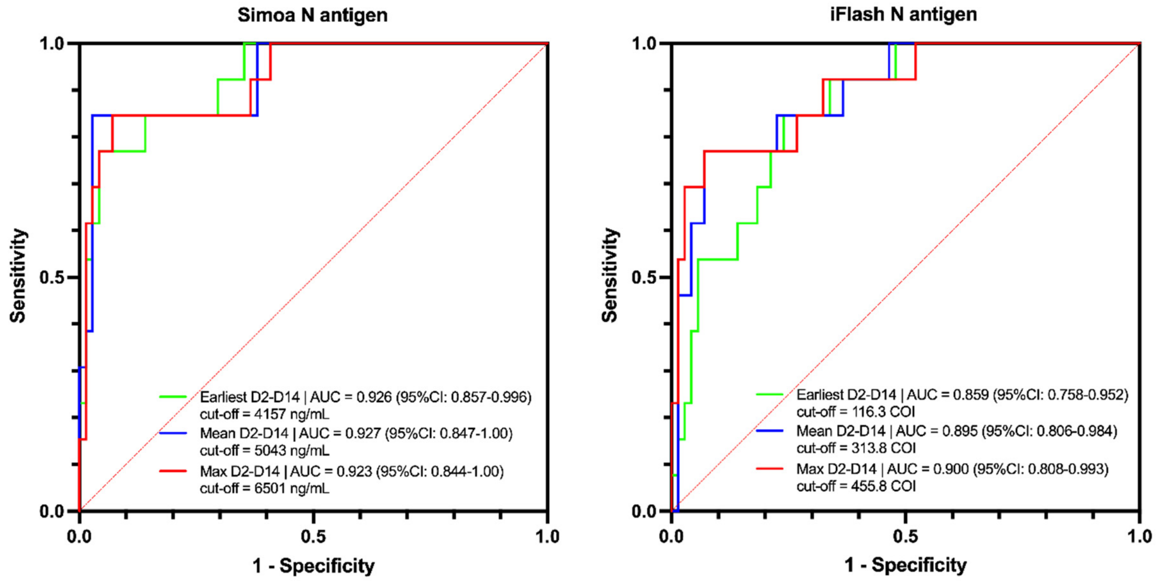

<u>Supplemental Figure S3:</u> Correlation between the Simoa and the iFlash N antigen assays. The grey dotted lines represent the cut-off determined by the ROC curves and the black dotted line on the iFlash axis represent the cut-off of the manufacturer. NPA, negative percentage agreement; PPA, positive percentage agreement.

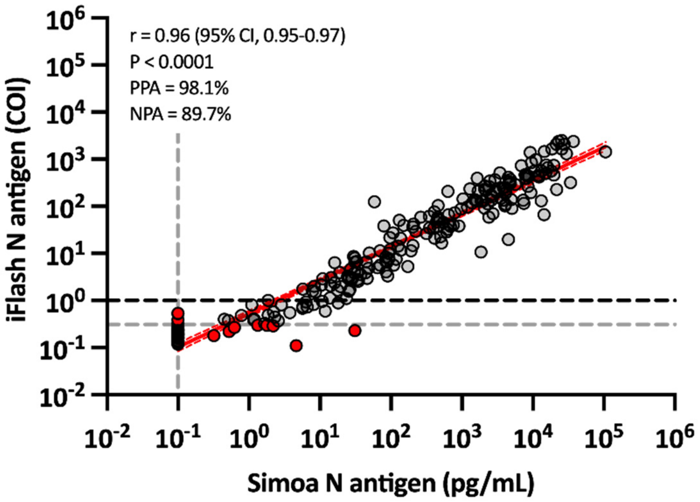

<u>Supplemental Figure S4:</u> Kinetics of antigenemia and SARS-CoV-2 Spike IgG antibodies since the onset of symptoms in non-severe and severe patients determined according to the WHO clinical progression scale.(20) The continuous orange line correspond to the severity cut-off of the Simoa assay and the green line to the iFlash assay, as found by ROC curves analyses. The purple dotted lines correspond to the positivity cut-off of the SARS-CoV-2 Spike IgG assay. The continuous red and blue lines correspond to antigen kinetics in severe and non-severe patients. The dotted red and blue lines correspond to the kinetics of SARS-CoV-2 Spike IgG in severe and non-severe patients. Only patients with symptoms and negative for IgG directed against the Spike protein at inclusion were included in this representation.

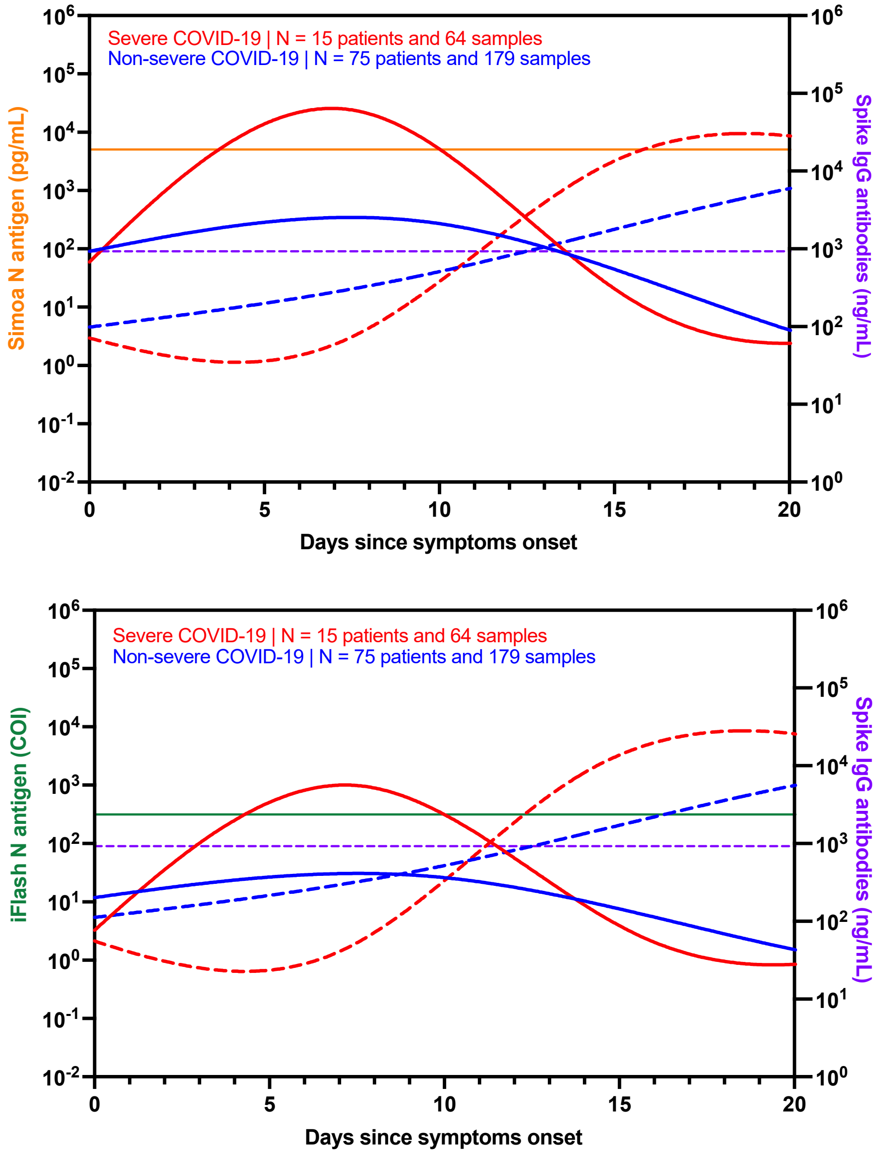

Supplemental Figure S5: SARS-CoV-2 Spike IgG results in serum in severe versus non-severe patients. The grey dotted lines on the Y-axis correspond to the positivity cut-off of the assay.

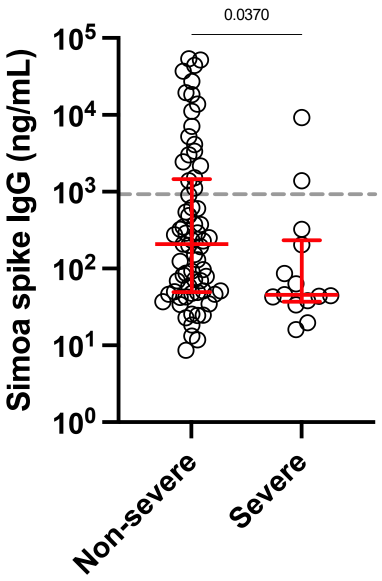

STARD checklist:

Section & Topic	No	Item	Reported on page #
**TITLE OR ABSTRACT**			
	**1**	Identification as a study of diagnostic accuracy using at least one measure of accuracy (such as sensitivity, specificity, predictive values, or AUC)	2
**ABSTRACT**			
	**2**	Structured summary of study design, methods, results, and conclusions (for specific guidance, see STARD for Abstracts)	2
**INTRODUCTION**			
	**3**	Scientific and clinical background, including the intended use and clinical role of the index test	3-4
	**4**	Study objectives and hypotheses	4
**METHODS**			
Study design	**5**	Whether data collection was planned before the index test and reference standard were performed (prospective study) or after (retrospective study)	4-5
Participants	**6**	Eligibility criteria	4
	**7**	On what basis potentially eligible participants were identified (such as symptoms, results from previous tests, inclusion in registry)	4-5
	**8**	Where and when potentially eligible participants were identified (setting, location and dates)	4-5
	**9**	Whether participants formed a consecutive, random or convenience series	4-5
Test methods	**10a**	Index test, in sufficient detail to allow replication	6-7
	**10b**	Reference standard, in sufficient detail to allow replication	6-7
	**11**	Rationale for choosing the reference standard (if alternatives exist)	3-4
	**12a**	Definition of and rationale for test positivity cut-offs or result categories of the index test, distinguishing pre-specified from exploratory	8
	**12b**	Definition of and rationale for test positivity cut-offs or result categories of the reference standard, distinguishing pre-specified from exploratory	8
	**13a**	Whether clinical information and reference standard results were available to the performers/readers of the index test	4-5
	**13b**	Whether clinical information and index test results were available to the assessors of the reference standard	4-5

Analysis	**14**	Methods for estimating or comparing measures of diagnostic accuracy	7-8
	**15**	How indeterminate index test or reference standard results were handled	7-8
	**16**	How missing data on the index test and reference standard were handled	7-8
	**17**	Any analyses of variability in diagnostic accuracy, distinguishing pre-specified from exploratory	7-8
	**18**	Intended sample size and how it was determined	/
**RESULTS**			
Participants	**19**	Flow of participants, using a diagram	5 and Figure 1
	**20**	Baseline demographic and clinical characteristics of participants	5 and Supplemental Table 1
	**21a**	Distribution of severity of disease in those with the target condition	5
	**21b**	Distribution of alternative diagnoses in those without the target condition	5
	**22**	Time interval and any clinical interventions between index test and reference standard	5 and Supplemental Figure 1
Test results	**23**	Cross tabulation of the index test results (or their distribution)<br>by the results of the reference standard	8-10
	**24**	Estimates of diagnostic accuracy and their precision (such as 95% confidence intervals)	8-10
	**25**	Any adverse events from performing the index test or the reference standard	/
**DISCUSSION**			
	**26**	Study limitations, including sources of potential bias, statistical uncertainty, and generalisability	13-14
	**27**	Implications for practice, including the intended use and clinical role of the index test	13-14
**OTHER INFORMATION**			
	**28**	Registration number and name of registry	/
	**29**	Where the full study protocol can be accessed	5
	**30**	Sources of funding and other support; role of funders	15
